# Quality of sleep and associated factors among medical interns in public universities in North Ethiopia

**DOI:** 10.3389/fpsyt.2025.1448028

**Published:** 2025-03-21

**Authors:** Dawit Afewerki Meles, Ashenafi Damte Ayele, Hagos Tsegaberhan, Tilahun Belete Mossie

**Affiliations:** ^1^ College of Medicine and Health Sciences, Aksum University, Aksum, Ethiopia; ^2^ School of Nursing, College of Health Sciences, Mekelle University, Mekelle, Ethiopia; ^3^ Department of Psychiatry, College of Medicine and Health Sciences, Bahir Dar University, Bahir Dar, Ethiopia

**Keywords:** quality of sleep, university, North Ethiopia, sleep hygiene, medical intern

## Abstract

**Objective:**

To assess the quality of sleep and associated factors among medical interns at public universities in North Ethiopia.

**Methods:**

We conducted a cross-sectional study among 259 medical interns using a structured, interviewer-administered questionnaire. We employed the Pittsburgh Sleep Quality Index (PSQI) to determine the quality of sleep.

**Results:**

Of the subjects, 72.6% were screened as having poor sleep quality. Anxiety [adjusted odds ratio (AOR) = 4.5, 95% CI: 1.93, 11.50; p < 0.001], poor sleep hygiene (AOR = 4.5, 95% CI: 1.4, 11.65; p < 0.001), current alcohol use (AOR = 3.85, 95% CI: 2.16, 6.89; p < 0.001), and current tobacco use (AOR = 2.4, 95% CI: 2.3, 25; p = 0.001) were significantly associated with poor quality of sleep.

**Conclusion:**

Poor quality of sleep is prevalent among medical interns. Addressing anxiety, and alcohol and tobacco use and enhancing the practice of sleep hygiene among medical interns requires significant attention.

## Introduction

Sleep is a complex behavioral state that is characterized by immobility or reduced behavioral responsiveness to external stimuli. Many researchers have concluded that sleep is essential for the growth and repair of the body and the improvement of cognitive functions such as motor skills learning, attention, and memory (1–4).

People spend approximately one-third of their life span asleep (2, 5). Although it varies from person to person, the average amount of sleep an adult needs is 7 to 8 hours per day (6, 7). Sleep disturbances are linked with several medical conditions including diabetes mellitus, heart disease, hypertension, and stroke. They are also related to a decline in cognitive performance and the development of mental health problems such as anxiety and depression (5, 8).

Investigators have concluded that due to reduced adult supervision, new social opportunities, a difficult curriculum, and various other extra-curricular activities, university students often have irregular sleep schedules and a higher rate of sleep deprivation and are affected by poor quality of sleep (9–11).

Because of the stressful nature of the field and demanding professional and academic requirements, medical students have poor quality of sleep (12). Compared with non-medical university students, the prevalence of poor quality of sleep is higher among medical students (13–16). Poor quality of sleep (PQOS) among medical interns is harmful; it affects their cognitive functions, performance, and physical health, leading to stress, depression, anxiety, and poor overall quality of life (17–20). Medical students have a worse quality of sleep compared to non-medical students and the general population. For example, a study conducted in Lithuania found that medical students had the highest prevalence of poor quality of sleep compared to other student groups (21).

Medical internship is the last year of study in medical school. During the internship, students face extended hours of shift, extensive workload, involvement in clinical decision-making, contact with diseases and death, and professional burdens, which contribute to the quality of sleep in this population. Many factors have been suggested to affect the quality of sleep (QOS) among medical interns. Socio-demographic factors (such as sex and marital status), academic performance, personal lifestyle, presence of stress, depression and anxiety, substance use, and chronic medical conditions have all been associated with the quality of sleep among medical interns (16, 22–33). In addition, a recent systematic review revealed that sleep disturbance has a significant relationship with suicidal behavior (34).

Although it has major gaps in its implementation, the Federal Democratic Republic of Ethiopia, Ministry of Health, has a school health program framework that guides universities to have programs to promote mental health; prevent mental, neurological, and substance use disorders; and provide counseling and behavioral management by a certified existing psychologist in campus clinics (35). However, there is no known system in Ethiopian higher education institutions to deal with them.

Even though poor quality of sleep is found to be remarkably high among pre-internship and intern medical students and is coupled with its negative impacts, to the best of the authors’ knowledge, there is a scarcity of research conducted about sleep quality among medical interns in East Africa including Ethiopia. Hence, the findings of this study will add to the existing shortage of knowledge about the problem. It may also help policymakers to implement the existing higher education-based health plan related to mental health and wellness. Medical schools in Ethiopia will also benefit in terms of enhancing the internship program. Furthermore, this study focused on assessing the quality of sleep and associated factors among medical interns in North Ethiopia.

## Methods

Study area and period: The study was conducted in public universities located in North Ethiopia. In this region, there are four public universities: Mekelle University, Adigrat University, Aksum University, and Raya University (36). Three of the four Universities—Mekelle, Adigrat, and Aksum universities—provide medical education. Mekelle University had 199 medical interns during the study period (37). Aksum University, located in the historical town of Aksum, had 28 medical interns (38). Adigrat University also had 54 medical interns (39). The study period was from June to July 2022.

Study design: An institution-based cross-sectional study was conducted.

Study population: All medical interns in public universities in North Ethiopia were the study population.

Eligibility criteria: Medical students who had a medical follow-up for a known mental health problem were excluded from the study since it may affect the main outcome of the study.

Sample size determination: All medical interns were included in the study.

Data collection procedures and tools: Data were collected from study subjects using pre-tested, interviewer-administered, structured, validated, and reliable tools. The questionnaire was originally developed in English and administered in Amharic, the official language in Ethiopia. The questionnaire included data about the quality of sleep, socio-demographic characteristics, sleep habit-related factors (daytime sleepiness and sleep hygiene), psychological factors, substance use, and chronic medical illness.

Data were collected by six Bachelor of Science degree-holder psychiatric nurses, and three master’s degree-holder mental health experts supervised the process. We trained the data collectors and supervisors for 1 day on the issues of the data collection process, application of the instrument, importance of consent, and how to maintain confidentiality.

We employed the Pittsburgh Sleep Quality Index (PSQI) to determine sleep quality. This tool has been widely used to examine sleep among various clinical, experimental, and normative samples (40). It also has good psychometric validity in screening for sleep problems among Ethiopian adults (41). We defined good quality of sleep as a cutoff score ≥5 on the PSQI.

The Epworth Sleepiness Scale (ESS) is the most widely used tool for assessing daytime sleepiness (42). It has excellent psychometric validity for screening daytime sleepiness among Ethiopian university students (43). A score of ≥11 on the ESS was considered indicative of daytime sleepiness. The Sleep Hygiene Index (SHI) is one of the most frequently used tools for the assessment of sleep hygiene. The SHI scale has demonstrated an adequate level of internal consistency among university students. A score of 27 or more on the SHI was defined as poor sleep hygiene (44).

The Perceived Stress Scale (PSS) was used to assess stress. It is a widely used tool for the assessment of perceived stress among students. According to a study conducted in Ethiopia, the PSS is a validated tool for the Ethiopian population Scores of 14–26 and >27 on PSS were defined as moderate and high levels of stress, respectively (45).

The Hospital Anxiety and Depression Scale (HADS) is an extensively used tool that assesses anxiety and depression symptoms (46). The HADS has been validated and widely used among the Ethiopian population. Scores of 11–21 on the HADS anxiety scale and 11–21 on the HADS depression scale were interpreted as having anxiety and depression, respectively (47).

*Study variables*: The quality of sleep was the outcome variable, whereas socio-demographic factors, psychological factors, substance use, and medical conditions were independent variables.

*Data processing, analysis, and quality control*: The data were coded, entered, and cleaned using Epi info version 3.1, and then, analysis was carried out using Statistical Package for Social Sciences (SPSS) version 25. Logistic regression was used to estimate adjusted odds ratios with a 95% confidence interval. Bivariate analysis was conducted to see the association of each independent variable with the dependent variable. Those factors with a p-value of less than 0.25 in the bivariate analysis were entered into multivariable logistic regression to identify the effect of independent variables on the dependent variable. The more relevant the variables we include, the better the understanding we will have of their potential interactions and impact on the multivariable analysis. It is a common practice to include variables that have a p-value less than 0.25 in the bivariate analysis, as these variables are considered to have a potential influence on the outcome variable and should be included in the multivariable model for further investigation. A p-value less than 0.05 in the final analysis was considered statistically significant.

The Hosmer–Lemeshow test showed a good fit (0.67). The internal consistency of the tool was acceptable at Cronbach’s alpha of 0.75. Additionally, potential multicollinearity was ruled out using the variance inflation factor, which was 1.58, so there was no multicollinearity effect. To ensure the quality of data, properly designed, validated, and reliable data collection instruments were used. The collected data were checked on a daily basis for completeness and consistency. The questionnaire was translated into Amharic language and back-translated into English by experts to ensure consistency. A pretest was conducted with 5% of medical interns who were at Mekelle University, Ayder Referral Hospital, and who were not included in the main study.

### Ethical considerations

Ethical clearance was obtained from Mekelle University, College of Health Sciences’s Institutional Review Board. Official letters of permission were obtained from the respective medical schools, and written consent was secured from each study participant. The right not to participate or to withdraw from the study was respected. Confidentiality and privacy were assured. Participants with higher scores on psychological tools were linked to nearby psychiatry clinics. The referral was considered after disclosing their scores on the scales. Based on their willingness, they were referred, but no personal identifier was used.

## Results

Socio-demographic characteristics: Among 259 participants, the mean age was 25.01 years (SD ± 1.96), and men accounted for 64.1%. Also, 93 (35.9%) live alone outside campus, and 71 (27.4%) live outside campus with a roommate. The mean grade point average (GPA) score was 3.08 (SD ± 0.33). The details are available in **Table 1**.

**Table 1:** Socio-demographic characteristics of medical interns in public universities of North Ethiopia, 2022.

Variable	Category	Number	Percent	Mean (SD)
Gender	Male	166	64.1%	
	Female	83	35.9%	
Age (years)	≤24	134	50.6%	25.01 (± 1.96)
	>24	128	49.4%	
Marital status	Single	249	96.1%	
	Married	10	3.9%	
Religion	Orthodox	164	63.3%	
	Muslim	32	12.4%	
	Protestant	46	17.8%	
	Catholic	8	3.1%	
	Others	9	3.5%	
Living accommodation	Campus dormitory	58	22.4%	
	Alone outside campus	93	35.9%	
	Outside campus with roommate	71	27.4%	
	With family	37	14.3%	
GPA (out of 4.0)	2.00–2.70	44	17%	3.08 (± 0.33)
	2.71–3.25	134	51.7%	
	3.26–3.75	77	29.7%	
	>3.75	4	1.5%	
Current ward	Medical ward	67	25.9%	
	Surgical ward	63	24.3%	
	Ob-gyn ward	63	24.3%	
	Pediatric ward	66	25.5%	

GPA, grade point average.

**Figure 1:**
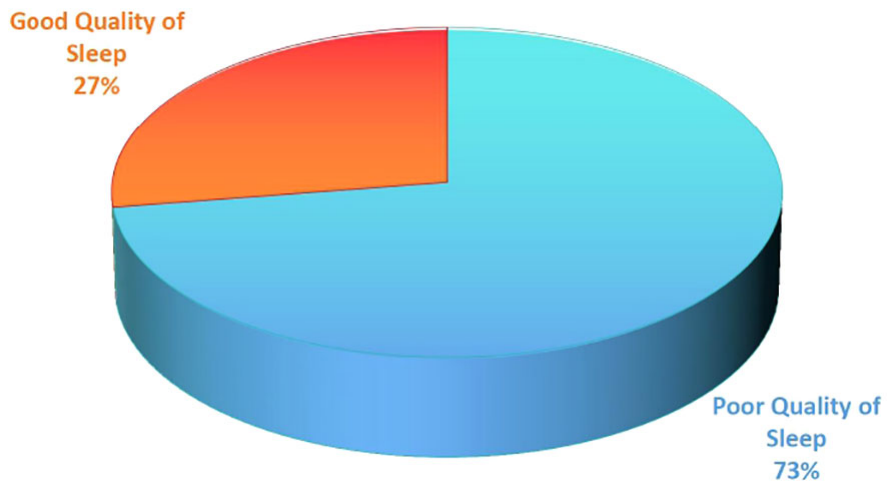
The prevalence of poor quality sleep among medical interns attending public universities, North Ethiopia.

Quality of sleep: The participants scored from 3 to 16 with a mean (SD) score of 7.84 (± 2.30). Approximately 72.6% (95% CI: 67.2%–78%) of the participants were screened as having poor sleep quality. The mean total time to fall asleep was 39:58 minutes (SD ± 21:53). Reported actual sleep hours ranged from 3:30 to 9:15 with a mean of 6:28 hours (SD ± 1:14). More than one-half of the participants 144 (55.6%) rated their overall subjective quality of sleep from fairly bad to very bad. See **Table 2** and **Figure 1**.

**Table 2:** PSQI sub-scales among medical interns of public universities of North Ethiopia, Ethiopia, 2022.

Variable	Category	Number	Percent
Sleep duration (hours)	<5.0	24	9.3%
	5.0–5.9	64	24.7%
	6.0–7.0	92	35.5%
	>7.0	79	30.5%
Sleep latency (minutes)	≤15	35	13.5%
	16–30	97	37.5%
	31–60	100	38.6%
	>60	27	10.4%
Daily dysfunction due to sleepiness	Never	68	26.3%
	<once a week	125	48.3%
	1–2 times a week	58	22.4%
	≥3 times a week	8	3.1%
Sleep efficiency (%)	>85%	210	81.1%
	75%–84%	48	18.1%
	65%–74%	2	0.8%
Sleep medication intake	Never	143	55.2%
	<once in a week	98	37.8%
	1–2 times a week	18	6.9%

PSQI, Pittsburgh Sleep Quality Index.

**Figure 2:**
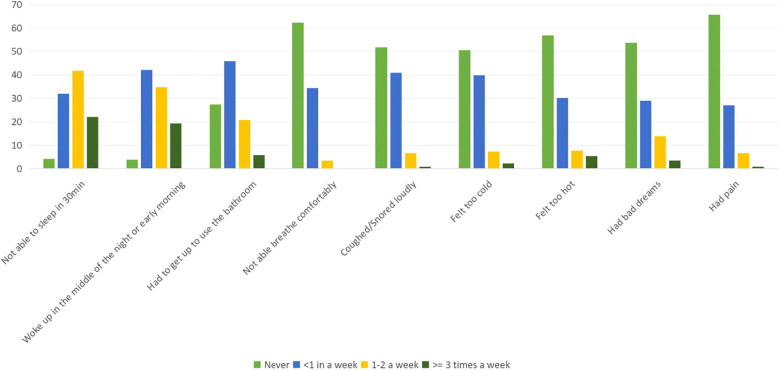
Percentage distribution of symptoms of quality of sleep among medical interns in public universities, North Ethiopia.

In the area of sleep disturbance, the study participants reported that 32% (n = 83) could not get to sleep within 30 minutes, and 42.1% (n = 109) reported waking up in the middle of the night or early morning awakening. Additionally, 45.9% (n = 119) had to get up to use the bathroom, 34.4% (n = 89) could not breathe comfortably, 40.9% (n = 106) coughed or snored loudly, 39.8% (n = 103) felt too cold, 30.1% (n = 78) felt too hot, 30.2 (n = 67) had bad dreams, and 29% (n = 75) had pain less than once a week. For more details, please see **Figure 2**.

Among the total participants, 60 (23.2%) experienced daytime sleepiness with a mean score of 7.09 (SD ± 4.03). Regarding the sleep hygiene practices according to the sleep hygiene index, 137 (52.9%) of the participants practiced poor sleep hygiene with a mean score of 25.46 (SD ± 8.05).

Psycho-social factors: According to the HADS, 21.6% and 24.3% had depression and anxiety, respectively. Among the study subjects, 71.8% and 9.6% experienced moderate and severe stress, respectively. In addition, in the 6 months prior to data collection, 157 (60.6%) of the study participants experienced stressful life events such as chronic medical illness, losing a friend, losing a family member, financial crisis, physical assault, friendship and relationship problems, and problems with authority.

Medical condition: Among the total study participants, 19 (7.3%) had known chronic medical illnesses, and 50 (19.3%) stated that they had a regular physical exercise practice.

Substance use: Those who had ever used substances included 79 (30.5%) for tobacco, 175 (67.6%) for alcohol, 37 (14.3%) for marijuana, 61 (23.6%) for khat, and 255 (98.5%) for caffeine drinks. The proportion of current users of substances was found to be 25.1% for tobacco, 56.0% for alcohol, 5.0% for marijuana, and 22% for khat.

### Factors associated with poor quality of sleep

The multivariable logistic regression analysis revealed that sleep hygiene, anxiety, current tobacco use, and current alcohol consumption were significantly associated with poor sleep quality. Medical interns with poor sleep hygiene practices were 4.5 times more likely to have poor sleep quality than those who have good sleep hygiene practices (4.5 95% CI: 1.4, 11.6). Similarly, study participants who have anxiety were 4.5 times more likely to be affected by poor quality of sleep than those not affected by anxiety (4.5 95% CI: 1.9, 11.65). According to substance use characteristics, medical interns who used tobacco currently were 2.4 times more likely to be affected by PQOS than those who did not currently use tobacco (2.4 95% CI: 2.3, 2.5). Finally, current alcohol consumers were found to be 3.8 times more likely to have PQOS than those who did not currently consume alcohol (3.8 95% CI: 2.159, 6.892). See **Table 3**.

**Table 3:** Multivariable logistic regression of independent factors with poor quality of sleep among medical interns in public universities in North Ethiopia, 2022.

Variable	Category	No. (%)	AOR (95% CI)	p-Value
Sleep hygiene	Good	122 (47.1)	1	<0.001
	Poor	137 (52.9)	4.5 (1.4, 11.65)	
Anxiety	No anxiety	123 (47.5)	1	<0.001
	Borderline anxiety	73 (28.2)	2.362 (1.182, 4.716)	
	Anxiety	63 (24.3)	4.5 (1.931, 11.504)	
Tobacco, current use	No	194 (74.9)	1	0.001
	Yes	65 (25.1)	2.4 (2.3, 25)	
Alcohol current use	No	114 (44.0)	1	<0.001
	Yes	145 (56.0)	3.858 (2.159, 6.892)	

AOR, adjusted odds ratio.

## Discussion

This study aimed to assess the quality of sleep among medical interns attending universities in North Ethiopia using the PSQI tool. It was found that the prevalence of poor quality of sleep among medical interns was 72.6% (95% CI: 67.2%–78%). Also, sleep hygiene, anxiety, alcohol use, and tobacco use were significantly associated with poor quality of sleep.

According to this study, 72.6% of the participants were screened as having poor quality of sleep. The higher prevalence in the current study may be attributed to the political instability and armed conflict in the area, which have the potential to affect the quality of sleep. Overall, the finding of the study agrees with previous findings. For instance, a study conducted in Pakistan reported that 77% of the participants had poor quality of sleep. Similarly, in Saudi Arabia, it was 74.2%. However, the findings of this study were higher than those of studies conducted in different settings including 65% in Bogotá, Colombia (28), 53.5% in Zanjan University, 51% in the USA (48), 42.3% in Brazil (17), and 32.5% in Nigeria (49). People in areas that reported variations in these findings may be influenced by the different socio-economic demands, cultural habits, academic demands, and population characteristics of the study subjects.

In the current study, one of the factors significantly associated with poor quality of sleep was sleep hygiene practices. The worse the sleep hygiene practice, the higher the odds of poor quality of sleep [adjusted odds ratio (AOR) 4.5 (95% CI, 1.4, 11.6)]. This finding is similar to that of prior studies conducted in Iran and Hong Kong, indicating that students with improper sleep hygiene practices reported worse sleep than students who practiced proper sleep hygiene. Sleep hygiene enhances the quality of sleep. Hence, its poor practice is associated with poor quality of sleep (23, 36).

Anxiety is also another factor that is significantly associated with poor quality of sleep in our study. Developing anxiety is related to higher odds of poor quality of sleep [AOR 4.5 (95% CI, 1.931, 11.504)]. An increase in symptoms of anxiety worsens the quality of sleep. This association can be viewed from the fact that one of the clinical features of anxiety is sleep problems. Previous studies conducted in Colombia and Estonia reported comparable results. Those studies also suggested that some sleep problems are indicative of underlying symptoms of anxiety (26, 28).

In this study, another factor associated with poor quality of sleep was the current consumption of alcohol. Participants who consumed alcohol had higher odds of poor quality of sleep than others who did not consume alcohol [AOR 3.85 (2.15, 6.89)]. This finding was congruent with a prior study conducted at the Pravara Institute of Medical Sciences that found that alcohol ingestion was significantly associated with poor quality of sleep. Such disturbances of sleep can be explained by the fact that current alcohol use is associated with shorter sleep onset, a decrease in the amount of rapid eye movement sleep, more stage 4 sleep in the first half of the night, an increase in waking during sleep, and an increase in electroencephalography delta time (31).

Current tobacco use was also a factor that showed a significant association with poor quality of sleep [AOR 2.4 (2.3, 25)]. This finding was in line with that of previous research conducted among Lebanese medical students indicating that increased cigarette use among university students was significantly associated with poor sleep patterns. With its stimulant effects and breathing-related complications of nicotine, smoking has a direct impact on sleep quality (29).

Moreover, poor quality of sleep was prevalent among medical interns. This implies that higher education institutions and other stakeholders should focus on modifiable factors, including sleep hygiene, anxiety, and substance use.

## Implications for future research

We suggest conducting further longitudinal studies to clearly establish a cause-and-effect relationship related to sleep disturbance and other factors that include academic activities, internship roles, and students’ performances and wellbeing. Recall bias may also affect the outcome. Other factors such as academic and clinical loads and dietary habits were not included in the study.

## Implications for policy and practice

The mental health, neurological, and substance use intervention packages planned to identify and manage mental health and substance use problems need to be implemented in higher education institutions. As sleep disturbance is related to mental health morbidity, it is advisable to consider sleep hygiene education, mental health support, or curriculum modifications for medical students.

## Limitations of the study

The cause-and-effect relationship cannot be established. Due to unstable security conditions, the study only included participants in the northern region, which may not allow generalization to other medical interns across the country. Additionally, there was ongoing conflict in North Ethiopia, which may have contributed to inflated prevalence.

## Data Availability

The raw data supporting the conclusions of this article will be made available by the authors, without undue reservation.
